# Effect of Membrane Materials and Operational Parameters on Performance and Energy Consumption of Oil/Water Emulsion Filtration

**DOI:** 10.3390/membranes11050370

**Published:** 2021-05-19

**Authors:** Nafiu Umar Barambu, Muhammad Roil Bilad, Nurul Huda, Nik Abdul Hadi Md Nordin, Mohamad Azmi Bustam, Aris Doyan, Jumardi Roslan

**Affiliations:** 1 Department of Chemical Engineering, Universiti Teknologi PETRONAS, Seri Iskandar 32610, Perak, Malaysia; barambunafiu@gmail.com (N.U.B.); nahadi.sapiaa@utp.edu.my (N.A.H.M.N.); azmibustam@utp.edu.my (M.A.B.); 2 Faculty of Applied Science and Education, Universitas Pendidikan Mandalika (UNDIKMA), Jl. Pemuda No. 59A, Mataram 83126, Indonesia; 3 Faculty of Food Science and Nutrition, Universiti Malaysia Sabah, Jalan UMS, Kota Kinabalu 88400, Sabah, Malaysia; jumardi@ums.edu.my; 4 Department of Food Science and Technology, Faculty of Agriculture, Universitas Sebelas Maret, Surakarta 57126, Indonesia; 5 Master of Science Education Program, University of Mataram, Jl. Majapahit No. 62, Mataram 83125, Indonesia; aris_doyan@unram.ac.id

**Keywords:** oil/water emulsion, membrane fouling, hydraulic resistance, membrane development, energy consumption

## Abstract

Membrane technology is one of reliable options for treatment of oil/water emulsion. It is highly attractive because of its effectiveness in separating fine oil droplets of <2 µm sizes, which is highly challenging for other processes. However, the progress for its widespread implementations is still highly restricted by membrane fouling. Most of the earlier studies have demonstrated the promise of achieving more sustained filtration via membrane material developments. This study addresses issues beyond membrane development by assessing the impact of membrane material (blend of polysulfone, PSF and polyethylene glycol, PEG), operational pressure, and crude oil concentration on the filtration performance of oil/water emulsion. The filtration data were then used to project the pumping energy for a full-scale system. Results show that fouling resistant membrane offered high oil/water emulsion permeability, which translated into a low energy consumption. The oil/water emulsion permeability was improved by three-fold from 45 ± 0 to 139 ± 1 L/(m^2^ h bar) for PSF/PEG-0 membrane in comparison to the most optimum one of PSF/PEG-60. It corresponded to an energy saving of up to ~66%. The pumping energy could further be reduced from 27.0 to 7.6 Wh/m^3^ by operation under ultra-low pressure from 0.2 to 0.05 bar. Sustainable permeability could be achieved when treating 1000 ppm oil/water emulsion, but severe membrane fouling was observed when treating emulsion containing crude oils of >3000 ppm to a point of no flux.

## 1. Introduction

Efficient management of industrial oil/water emulsion wastewater has been a challenge due to poor efficiency of conventional treatment methods [1,2,3], especially when treating emulsion with fine oil droplet sizes of <2 µm [4]. Dissolved air floatation and many other conventional methods are capital intensive and involve generation of secondary waste [5,6]. Those limitations hinder their continuous industrial implementation [7,8]. On the other hand, pressure-driven membrane-based technology has been explored for handling oil/water emulsion due to its economic and environmental advantages [5,9,10].

The pressure driven membrane process involves a size exclusion mechanism to separate oil droplets from the emulsion. It has been proven effective for separating fine oil droplets [11], which led to intensive research and developments. However, many studies were only focused on membrane material development without addressing many other factors in oil/water emulsion treatment, like energy consumption. Zhu et al. [12] reported up to ~3.7-fold performance improvement by developing PSF membrane via a combination of vapor and nonsolvent induced phase separation. By prolonging humid air exposure time after casting the polymer film from 0 to 5 min, both the structure and surface chemistry of the membrane shifted gradually. They reported that a top immobile layer polymer matric (formed in the earlier stage of exposure to humid air) restricted the leaching of additive poly(ethylene glycol) methacrylate from the membrane matrix. Similarly, Mat Nawi et al. [13] developed a polyvinylidene fluoride membrane using PEG additive using the same fabrication method. Upon extending the exposure time from 0 to 30 min, up to 35% enhancement of permeability for oil/water emulsion filtration was achieved.

The overall economic performance of the membrane-based process is strongly affected by the energy input [14], mainly for feed pumping in a crossflow filtration system [15,16]. Therefore, apart from the hydraulic performance, the energy assessment of the membrane process remains vital for its continued widespread adoption [14,17]. The energy consumption in crossflow filtration is strongly affected by the applied parameters, such as the fouling propensity of the selected membrane material, applied crossflow velocity, pressure, and feed oil concentration [18,19,20]. Membrane development offers the most adopted approaches to restrict the increase in hydraulic resistance [21,22], hence lowering the energy consumption due to low crossflow velocity and low applied pressure [23,24,25]. As such, it enhances the economic viability of the system by lowering the energy footprint [26]. Membrane material developments were generally done by transforming the surface chemistry from hydrophobic to hydrophilic [12,27], which resulted in a substantial increment in hydraulic performance. However, limited studies are available on the evaluation of those developed membranes to a more practical level, i.e., evaluating the effect of operational parameters and energy consumption.

Several reports addressed the effect of filtration parameters on the energy input of oil/water emulsion filtration [28,29,30]. Li et al. [31] investigated the effect of rotating disk with vanes on energy consumption for linseed oil/water emulsion filtration. The system recorded up to ~140% energy saving against the smooth disk. The smooth disk and disk with vanes exhibited 1070 and 445 kWh/m^3^ specific energy consumption, respectively. Substantial reduction in energy input was achieved thanks to the improved hydrodynamic to suppress membrane fouling. Krstić et al. [30] reported energy saving of up to ~40% using the static mixer as turbulence promoter in cutting oil/water emulsion filtration. Similarly, Vatai et al. [29] also reported tremendous energy saving, which was attributed to the hydrodynamic effect induced by the static mixer and the sparged air bubbles. Scott et al. [32] reported the effect of corrugation angle on energy consumption of crossflow water/oil emulsion filtration. The corrugated membrane demonstrated up to 88% energy saving against the reference. Most of the reports that address the energy input for oil/water emulsion filtration focus on hydrodynamic enhancement, and less so on the impact of membrane material and operational parameters.

In our previous report [33], a hydrophilic polysulfone (PSF) membrane was developed by restricting the leaching of PEG additive during phase inversion. By adjusting the exposure time of the cast film under humid air before immersion into the water bath, membrane structure and chemistry were altered resulting in an improvement by >2-fold of the performances. However, the ultimate effects on the energy consumption together with other operational parameters such as transmembrane pressure (Δ*P*) and feed oil concentration were not reported. To demonstrate the benefit of membrane material development, an assessment of its impacts on the overall energy consumption is required and thus addressed in the current report.

In this study, the effects of operational parameters on oil/water emulsion filtrations, namely membrane materials, Δ*P* (or crossflow velocity), and feed oil concentration on the hydraulic performances were studied. The filtration data were then used as a basis to estimate the energy consumption of a full-scale set-up by using a projected full-scale plate-and-frame module. The scope of this study extends beyond what was reported in our earlier work [33] that merely focused on membrane material development.

## 2. Materials and Methods

### 2.1. Membrane Preparation and Characterization

The details of membrane preparation and characterization are available in our previous study [33]. Briefly, 18 wt% polysulfone (PSF, Mw = 35,000 g/mol, Sigma Aldrich, St Louis, MO, USA), 1 wt% PEG (Mw = 10,000 g/mol, Sigma Aldrich, St Louis, MO, USA) and 0.1 wt% lithium chloride (LiCl, Mw = 42.38 g/mol, ACROS Organics, Geel, Belgium) were loaded into 80.9 wt% dimethylacetamide (DMAc, 99.8% purity, Sigma Aldrich, St Louis, MO, USA). The mixture was stirred at 60 °C to form a homogeneous dope solution. The resulting dope solution was cast atop three separate nonwoven supports (Novatexx 24413, Freedenberg-filter, Weinheim, Germany) at 0.22 mm wet casting thickness. The casted films were then exposed to air (of ~70% relative humidity) for 0, 30, and 60 s before immersion into water bath. The resulting membranes were denoted as PSF/PEG-0, PSF/PEG-30, and PSF/PEG-60 according to the exposure time durations. The trials on preparing membranes with >60 s time gaps were performed. However, they resulted in membranes with poor mechanical strength that could not be used for filtration due to poor rejection and delamination of the PSF film from the nonwoven support. The pore size distribution, thickness, and water contact angle of the membranes were measured using a capillary flow porometer (Porolux 1000, Berlin, Germany), micrometer (MDC-1, Mitutoyo, Japan), and goniometer (Ramé-Hart 260, New Jersey, USA) respectively.

### 2.2. Oil/Water Emulsion Preparation

The synthetic oil/water emulsion feeds with oil concentrations of 1000, 3000 and 5000 ppm were prepared. The feed consisted of sodium dodecyl sulfate (SDS, 98% purity, Sigma Aldrich, USA), real crude oil (obtained from a crude oil well in Malaysia), and distilled water as the surfactant, the oil, and the water, respectively. For each oil concentration, a 1:99 w/w SDS to oil ratio was first prepared and used to form a stock stable emulsion. Subsequently, 0.1, 0.3, and 0.5% of the prepared oil/SDS mixture were mixed with one liter of water separately at a stirring rate of 3500 rpm for 24 h to obtain the 1000, 3000, and 5000 ppm oil/water emulsion, respectively. A small volume of feed samples was later analyzed using a particle size and zeta potential analyzer (Zetasizer Nano ZSP, Malvern, UK) to map the oil droplet size distribution.

### 2.3. Filtration Test

**Figure 1:**
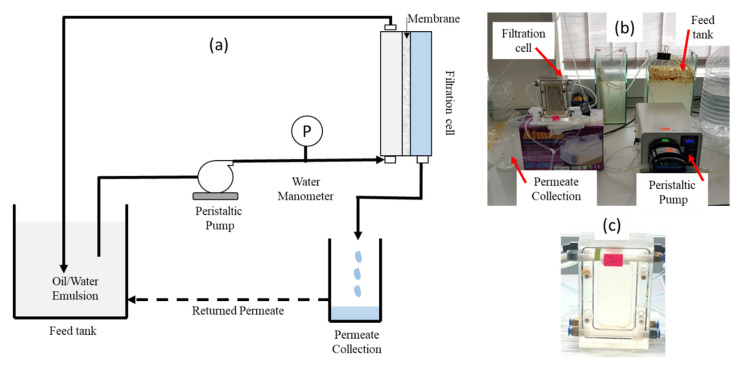
(**a**) Illustration and (**b**) pictures of the filtration set-up (**c**) and picture of the home-made filtration cell.

Each membrane sample was cut at dimension of 8.8 (length) × 4.2 cm (width) corresponding to an effective membrane area of 0.0037 m^2^ and was installed into the chamber of the crossflow filtration set-up illustrated in Figure 1. The filtration cell was made from poly(methyl methacrylate) consisting of a flow channel with the cross section area of 4.2 cm in width and 0.2 cm in depth filled with a net polyethylene spacer in the permeate side only. The crossflow velocity was obtained from the volumetric velocity divided by the cross-section flow area. Briefly, a peristaltic pump was used to circulate the feed at the desired velocity. The water manometer was used to monitor the feed pressure and the Δ*P* was regulated by adjusting the rotation speed of the peristaltic pump (Figure 1B). Each filtration test was done in a full-recycle system to maintain the condition of the feed over the filtration time with 5 L of feed emulsion in the feed tank. The permeate was returned manually to the feed tank after volume measurement. The full-recycle method was applied since the concentration filtration as in the actual application was technically difficult to conduct using a very small membrane area. The permeate volume was measured every 10 min in which the permeate collector was replaced for collection of the subsequent 10 min filtration. During the following permeate collection, the volume of permeate from previous cycle was then measured and subsequently returned to the feed tank, and so on. A full-recycle filtration of 3000 ppm of oil/water emulsion can represent a batch full-scale concentration of much lower oil in the feed to reach a final concentration of 3000 ppm (i.e., from 100 to 3000 ppm).

Firstly, the membrane was compacted under Δ*P* of 0.2 bar for 60 min by filtration of clean water, after which an almost stable permeability was achieved. Subsequently, the clean water permeability was obtained from 30 min of clean water filtration. Thereafter, the feed was switched to the oil/water emulsion detailed in Section 2.2. The volume of permeate was evaluated every 10 min for 90 min. The collected permeate was returned to the feed tank to maintain the feed condition. After that, the feed was switched back to DI water for 10 min for foulant flushing. At the same time, the permeability of the fouled membrane was also measured. The filtration of oil/water emulsion followed by water flushing was considered as one filtration cycle. The filtration was continued for four more cycles resulting in a total of five cycles per test. Each test was repeated two times and the results are presented as average ± standard deviation. The repetitions of the filtration tests were done using the same membrane coupon to avoid large variation between membrane samples. After completion of each filtration, the fouled membrane was cleaned physically by gentle wiping with soft sponge for about 10 min, followed by immersion in 1000 ppm of sodium hypochlorite solution for one hour. In all tests, the clean permeability of the membrane was almost fully restored (>95% of the pristine value).

Three series of filtration tests were conducted. Firstly, effect of membrane properties was assessed at Δ*P* of 0.2 bar using 1000 ppm oil/water emulsion as the feed from which PSF/PEG-60 was selected for further tests. Secondly, effect of Δ*P* on the hydraulic performance of PSF/PEG-60 membrane was evaluated for filtration of 1000 ppm oil/water emulsion feed, from which Δ*P* of 0.2 bar showed the best performance. The Δ*P*s were set at 0.05, 0.10, 0.15 and 0.20 bar, corresponding to crossflow velocity of 0.5, 1.0, 3.9 and 5.5 cm/s, respectively. The range of cross-flow velocity applied in this study is within the one applied in commercial full-scale module [34]. It is worth noting that the Δ*P* was—due to set-up limitation—altered by changing the crossflow velocity by changing the rotation speed of the peristaltic pump (Figure 1B). It has to be noted that the pressure–velocity relationship in our filtration system can have an overlapping effect requiring an independent investigation. In our system, higher cross-flow velocities were required for filtration at higher Δ*P*s. Lastly, filtration performance of the PSF/PEG-60 membrane was evaluated using 1000, 3000 ppm, and 5000 ppm oil/water emulsion feeds at Δ*P* of 0.2 bar.

The clean water and the oil/water emulsion permeabilities (*L*, L/(m^2^ h bar)) were calculated using Equation (1), while oil rejection (R, %) was calculated using Equation (2).

(1) $$L=\frac{\Delta V}{A\;\Delta t\;\Delta P\;}$$

(2) $$R=\left(1-\frac{C_{p}}{C_{f}}\right)\times 100$$

where Δ*V* is the permeate volume collected (L), Δ*t* is the filtration time (h), *A* is the membrane effective area (0.0037 m^2^), *C_p_* is the permeate oil concentration obtained from measurement (ppm), and *C_f_* is the feed oil concentration obtained from measurement (ppm). The *C_p_* was evaluated using a UV-VIS spectrometer (Shimadzu UV-2600, Kyoto, Japan) at a wavelength of 227 nm.

### 2.4. Estimation of Energy Consumption

To estimate the energy consumption, the projected full-scale set up was assumed as a simple system, in which only the feed pump acted as the energy consuming component. Therefore, the work done by the pump ($W_{p},$ J/kg) was estimated using Equation (3). The full-scale module was assumed to be horizontally aligned to a plate and frame module composed of a stack of horizontal flat sheet membranes. Each module panel has a panel width and a height of 1.0 and 2.0 m in a plate and frame module configuration. The gap between the two adjacent panels was set to be 2 × 10^−3^ m. The crossflow velocity and the Δ*P* (Pa) were set the same as the ones applied in the experiments. The plate-and-frame module was selected because it more resembled the filtration set-up used in the present study. Based on the available information, the friction loss along the module was also included in the estimation by employing the Blasius equation [35]. The specific energy consumption per unit permeate produced (*E_P_*, kWh/m) was estimated using Equation (4).

(3) $$W_{p}=\frac{\frac{P_{1}}{\rho }+\frac{V^{2}}{2}+F}{3,600,000}$$

(4) $$E_{p}=\frac{ṁW_{p}}{V_{P}}$$

where $P_{1}$ is the feed pressure (according to the one applied in the test), ρ is the density of water (1000 kg/m^3^), 𝒱 is the crossflow velocity (m/s), *F* is the laminar flow friction loss (m^2^/s^2^), ṁ is the mass rate (kg/s), and $V_{P}$ is the volumetric rate of permeate produced by the membrane (m^3^/h).

## 3. Results and Discussion

### 3.1. Membrane and Oil/Water Emulsion Properties

The membranes’ properties are summarized in Table 1. The pore size gradually decreased from PSF/PEG-0 to PSF/PEG-30, and PSF/PEG-60 as follows from 0.126, to 0.057, and 0.032 µm, respectively. It can be attributed to the rapid solidification of the cast film during the humid air exposure leading to the faster demixing as also detailed elsewhere [12,36]. It could be visually observed that a short exposure to humid air changed the color of the film from transparent to white. The solidification of the top layer of the film blocked the outflow of PEG during the immersion in the nonsolvent, resulting in higher PEG entrapment near the membrane surface and enhancement of PEG density near the surface which lowered the contact angle, as also reported earlier [13]. The membrane thickness gradually increased as the membrane was developed due to the increased PEG surface density, but would only slightly affect the hydraulic performance due to the asymmetric nature of the membranes [37]. As shown in Table 1, all membranes were hydrophilic (contact angles of < 90°) when evaluated using pure water. Indeed, the contact angle of the actual feed can provide richer information on the surface interaction between the feed and the membrane surface. However, we believe that water contact angle data presented in Table 1 provided sufficient information on the emulsion/membrane interaction because the feeds were oil-in-water systems, in which water is the dominant phase. Under this condition, the pure water and the oil/water emulsion contact angles were expected to be about the same.

Overall, the structural and chemical properties of PSF/PEG-60 lead to the highest clean water permeability of 502 ± 9 L/(m^2^ h bar), significantly higher than PSF/PEG-30 and PSF/PEG-0 with respective clean water permeabilities of 365 ± 7 to 329 ± 8 L/(m^2^ h bar). Detailed discussion on the correlation between fabrication methods and the resulting membrane properties have been reported in our earlier work [33].

**Figure 2:**
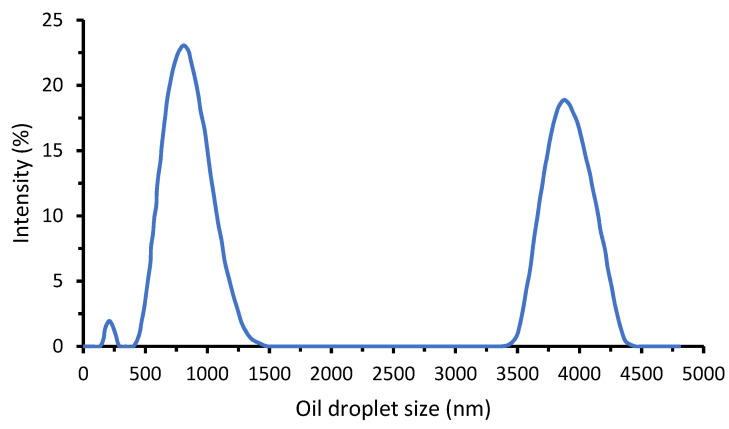
Size distribution of oil droplet in oil/water emulsion sample.

Figure 2 shows the distribution of oil droplets in oil/water emulsion sample. The synthesized oil/water emulsion was found stable during the experiment duration for about 2 months without any sign of settling or oil flotation. The intensity distribution is multimodal with the dominant sizes of 0.25, 0.01 and 4.0 µm. Judging from the particle size distribution and by comparing with the pore size of the membrane, the two largest clusters of droplet size distribution were expected to be fully retained by the membrane.

### 3.2. Oil/Water Emulsion Filtration

#### 3.2.1. Effect of Membrane Material

**Figure 3:**
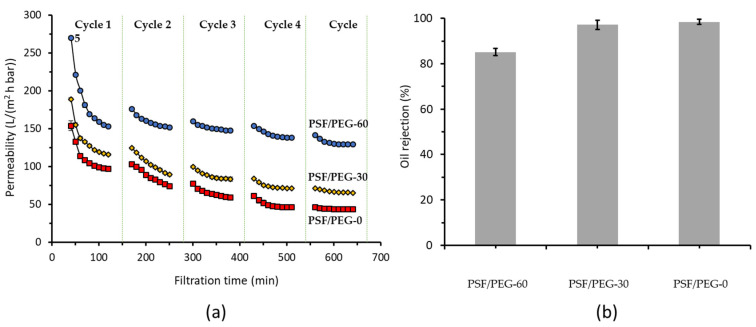
Effect of membrane material on the filtration performance (**a**) and oil rejection (**b**). The tests were conducted at a constant transmembrane pressure of 0.2 bar for treating 1000 ppm oil/water emulsion feed. The first 30 min of filtration in the first cycle was done for clean water permeability measurement.

Figure 3 shows the filtration performance of the membranes. The effect of membrane materials on the permeability of oil/water emulsion filtration and oil rejection can be related to the properties summarized in Table 1. PSF/PEG-60 outperformed the rest by showing the highest permeability and rejection. Even after the fifth filtration cycle, the PSF/PEG-60 showed permeability of up to 139 ± 1 L/(m^2^ h bar). Whereas PSF/PEG-30, PSF/PEG-0 exhibited permeabilities of 70 ± 0 L/(m^2^ h bar) and 45 ± 0L/(m^2^ h bar), respectively. Similarly, another report showed an enhanced throughput by up to ~55% by developing polyvinylidene fluoride membranes [13]. Dehban et al. [38] and Zhu et al. [12] also reported a lower filtration resistance for optimally developed membranes.

The results confirm that good membrane development helped in enhancing filterability of oil/water emulsion. As shown in Figure 3, the PSF/PEG-60 exhibited the higher permeability due to the improved membrane surface chemistry. Membrane development via the vapor induced phase separation method enhanced surface hydrophilicity, which promoted a hydration that facilitated transport of water [39]. Surface water hydration also favored the oil rejection as depicted in Figure 3.

Improvement in the PEG surface density via membrane development played two important roles in enhancing the oil rejection performance (Figure 3b). Firstly, it improved the surface chemistry by transforming the surface to hydrophilic, as such restricted interaction between oil droplet and membrane surface. Secondly, it reduced the surface pore size, and enlarged the size range of rejected oil droplets. Thanks to its best filtration performance, only PSF/PEG-60 was used in the study on the effect of crossflow velocity and the effect of oil concentration. It is worth noting that sorption of oil by PSF can play an important role in oil removal [40], especially in the beginning of the filtration until it reached the maximum sorption capacity.

#### 3.2.2. Effect of Transmembrane Pressure

**Figure 4:**
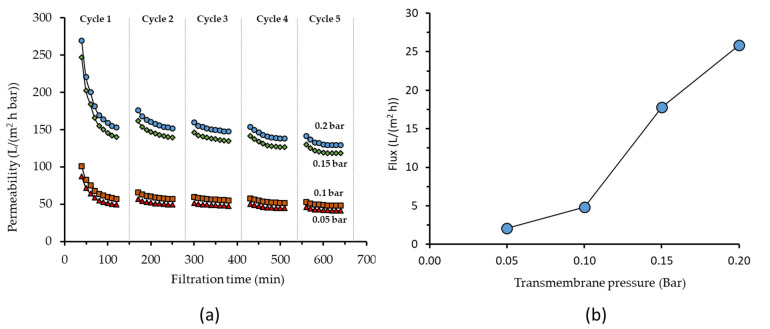
Effect of transmembrane pressure (TMP) on (**a**) permeability as function of filtration time and (**b**) flux from the final reading of the last filtration cycle of PSF/PEG-60 membrane treating 1000 ppm oil/water emulsion under various TMP. The first 30 min of filtration in the first cycle was done for clean water permeability measurement.

Figure 4a shows the effect of the ΔP on the permeability of 1000 ppm oil/water emulsion. Higher ΔP increased the system throughput. Filtrations under ΔPs of 0.2, 0.15, 0.1 and 0.05 bar resulted in permeabilities of 139.46 ± 0.81 to 118.5 ± 0.2 to 48.3 ± 0.2, and 42 ± 0 L/(m^2^ h bar), respectively. The decreasing trend of permeability at lower ΔP was also reported elsewhere [41].

The increase in permeability at higher ΔP can be explained by the dual roles of crossflow velocity in affecting the ΔP and in scouring-off the foulant for membrane fouling control. For filtration of clean water under no fouling, permeability is theoretically constant at any given ΔP. However, when treating fouling prone feed such as oil/water emulsion, membrane fouling played an important role. Higher crossflow velocity at higher ΔP resulted in better fouling control. At low ΔP, the crossflow velocity was too-low and less effective in scouring-off the foulant. Consistent results have been reported on the efficacy of crossflow velocity in controlling membrane fouling, including for oil/water emulsion filtration [42,43,44,45]. On the other hand, high crossflow velocity also associates with high pumping energy as depicted in Equation (4). The contribution of concentration polarization in diminishing permeability is considered low because of a low flux and a low applied pressure.

One of the objectives to assess the impact of ΔP on oil/water emulsion filtration is to lower the pumping energy. In a recent report [46], it was mentioned that filtration under low ΔPs substantially affected membrane compaction and permeability. As a result, the filtration fluxes under variable pressures below 0.1 bar were not effective for the treatment of laundry wastewater. A different trend was observed in this study in which the flux increases at higher ΔPs, as shown in Figure 4b. It is worth noting that even though there was higher plateau flux, the initial flux decline is clearly more pronounced for more hydrophilic membranes (Figure 3) and when testing at lower TMP (Figure 4), compared to less hydrophilic membranes and higher TMP. The rapid decline in the earlier stage of filtration can be attributed to a higher drag force that carried the oil droplet toward the pore mouth by the permeate flow at higher flux.

#### 3.2.3. Effect of Oil Concentration

**Figure 5:**
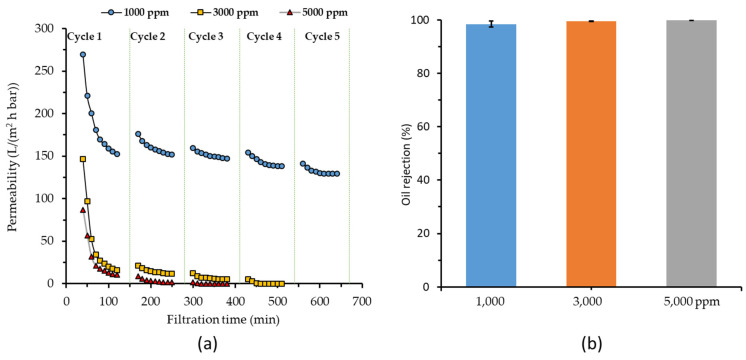
Effect of feed oil concentration on (**a**) hydraulic performance and (**b**) oil rejection for filtration of 1000 ppm oil/water emulsion feed using PSF/PEG-60 membrane evaluated at a constant transmembrane pressure of 0.2 bar.

Figure 5a shows the severe impact of high oil concentration on hydraulic performance. For oil concentrations of 3000 and 5000 ppm, the membrane fouling was highly irreversible. Permeate was only obtained until cycles 3 and 4 for 3000 and 5000 ppm, respectively. Beyond those cycles, the membrane surface was fully blocked leaving no available open pores for water permeation. Under such conditions, other means of membrane cleaning (on top of water flushing) are necessary to sustain the filtration. These results demonstrate the significance of oil concentration in oil/water emulsion feed on the buildup of the hydraulic resistance and more oil droplets/membrane surface interaction. Too-high oil concentrations diminished the hydraulic performances. The same finding was reported elsewhere [47], in which severe membrane fouling completely blocks the membrane pore resulting in zero permeation.

Interestingly, the sustained operation was demonstrated for filtration of 1000 ppm of oil/emulsion feed. A permeability of around 150 L/(m^2^ h bar) was achieved at the end of the first cycle and could be maintained toward the end of the fifth cycle. For higher oil concentrations, almost all permeability diminished within the first cycle and could not be restored through water flushing in the subsequent cycles. The finding suggests the presence of critical oil concentration—in between 1000 and 3000 ppm—below which a sustained filtration could be achieved. Below this critical oil concentration, the filtration would be sustained without minimum cleaning. Practically, the setting for concentration level should be done slightly under the critical concentration to achieve both sustainable filtration and high permeate recovery. Despite showing slow decline of permeability during the five filtration cycles, a slow decline in permeability would still be expected in a long-term operation, possibly requiring a chemical cleaning. The detailed relationship between the crude oil concentration in the emulsion and sustainable filtration performance can be an interesting topic of a follow up study.

Figure 5b shows almost complete rejection of oil irrespective of the oil concentration in the feed solutions. As discussed in Section 3.2.1, good oil rejection was achieved by PSF/PEG-60 membrane thanks to its hydrophilic nature coupled with low mean flow pore size. At higher oil concentrations, full coverage of oil was expected on the membrane surface acting as an additional filtration cake layer that promotes better oil rejection.

### 3.3. Energy Consumption

**Figure 6:**
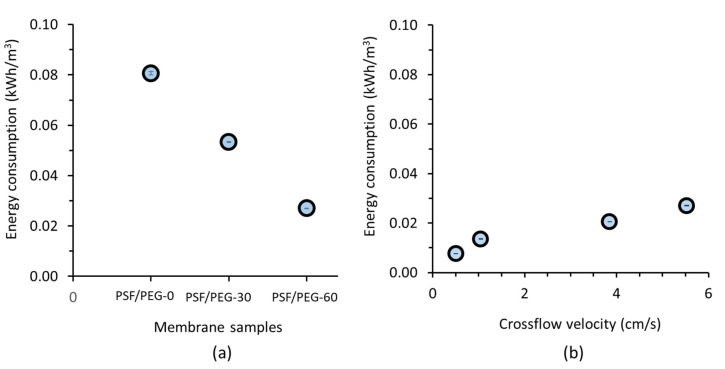
Specific energy consumption for filtration of 1000 ppm oil/water emulsion as a function of (**a**) applied membrane material and (**b**) energy consumption using PSF/PEG-60 membrane as a function of applied crossflow velocity using the permeability data at the final reading of the fifth filtration cycle.

**Figure 7:**
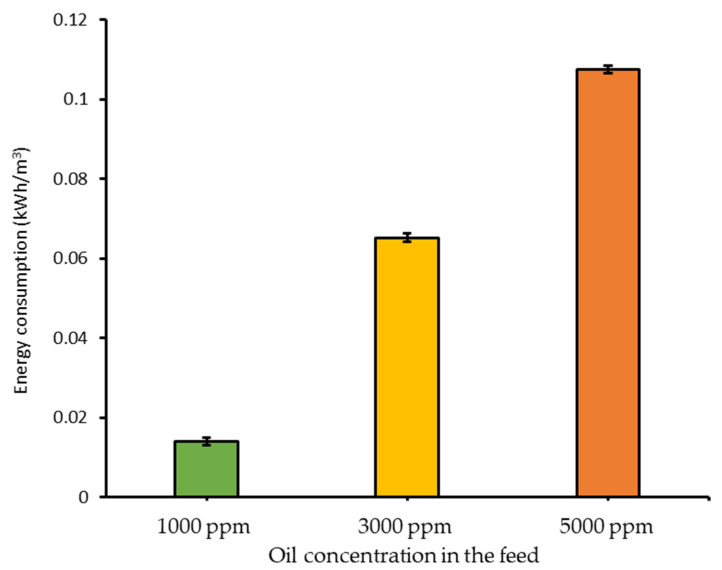
Effect of oil concentration in the feed solution on the specific pumping energy consumption using PSF/PEG-60 under transmembrane pressure of 0.2 bar. The energy consumption estimations were done using permeability data at the final reading of the first filtration cycle.

Figure 6 and Figure 7 show the effect of membrane material, operational ΔP, and oil concentration on the pumping energy. The specific energy consumptions in Figure 6 were obtained using the final permeability of the fifth cycle. Figure 6a suggests that the application of a membrane with low fouling and high permeability substantially reduced the pumping energy. By comparing the specific energy consumptions of PSF/PEG-0 and PSF/PEG-60 membranes, it can be seen that the latter shows >66% energy saving. The selected membrane for further exploration test (PSF/PEG-60) posed only 27.0 ± 0 Wh/m^3^ energy against 80.0 ± 0 and 53.0 ± 0 Wh/m^3^ exhibited by PSF/PEG-0 and PSF/PEG-30, respectively. This is a significant energy saving, especially when considering full-scale industrial applications. Nonetheless, one can argue that application of very low flux can inflate the membrane investment cost. However, due to decreasing trend of the membrane cost, a trade-off between low operational costs by using a large membrane area (operated at low flux) can offer the most cost-effective system.

Consistent reports have demonstrated the importance of membrane development and/or fouling control systems to achieve energy saving [12,36,48,49]. Krstić et al. [30] reported up to ~40% energy saving when applying a static mixer as turbulence promoter during cutting oil/water emulsion filtration. In another study, Li et al. [31] reported up to 58% energy saving when applying rotating disks with and without vanes for linseed oil/water emulsion filtration.

Figure 6b demonstrates the drawback of high crossflow velocity in increasing the pumping energy. The pumping energy is almost proportional to the applied crossflow velocity. The energy consumptions of 7.6, 13.6, 20.5, and 27.0 Wh/m^3^ were obtained for filtration under crossflow velocities of 0.50, 1.04, 3.85 and 5.53 cm/s, respectively. In the present study, the energy for cleaning was excluded but assumed to be the same for different applied crossflow velocities. In the real application, it might be different and must be optimized independently. This finding suggests the need for system optimization since there is a trade-off between the high membrane investment at low crossflow velocity (low-flux) but with low energy consumption, and vice versa. The optimum parameters need to be set to minimize the overall costs for oil/emulsion filtration. Similarly, Scott et al. [32] also reported a directly proportional increase in energy consumption with an increase in the crossflow velocity during crossflow filtration of water/oil emulsion feed.

As discussed in Section 3.2.3, oil concentration in the feed significantly affected the filtration performance as also reflected by the energy consumption (Figure 7). Due to the absence of permeation beyond the third cycle for the feeds with 3000 and 5000 ppm crude oil concentration, the energy estimations in Figure 7 were done using permeability data at the final reading of the first filtration cycle. Due to higher oil/water emulsion permeability at oil concentration of 1000 ppm, its energy consumption was almost one magnitude lower than the one with 5000 ppm crude oil.

The high energy consumption coupled with severe membrane fouling for emulsion with high crude oil concentration would substantially affect the implementation of the developed membrane in full-scale. Without additional means of membrane fouling controls, such as aeration, module design modification can be applied to allow a more sustained operation. Alternatively, simple crossflow membrane filtration can be applied for the concentration of crude oil up to the critical concentration (a point between 1000 and 3000 ppm), in which sustained operation can be achieved under low energy input (Figure 6). Subsequently, another more attractive process can be applied to achieve a higher permeate recovery, which is a two-stage approach proposed for the concentration of microalgae by combining membrane-based processes with others [50,51].

## 4. Conclusions

The membrane materials, operational ΔP, and crude oil concentration in the emulsion feed significantly affected the filtration performance of oil/water emulsion. From membrane development, the oil/water emulsion permeability was improved almost three-fold from 45 ± 0 to 139 ± 1 L/(m^2^ h bar) for PSF/PEG-0 (as reference) to the most optimum one of PSF/PEG-60. Such improvement corresponded to an energy saving of up to ~66%. Application of PSF/PEG-60 membrane was even more efficient under a low ΔP. Lowering the ∆*P* from 0.2 to 0.05 bar depleted the specific energy consumption from 27.0 to 7.6 Wh/m^3^. However, due to severe membrane fouling, no permeation was achieved beyond the third filtration cycle for filtration of oil/water emulsion containing crude oils of >3000 ppm. This finding suggests the implementation of multistages filtration incorporating control of fouling for feeds with higher crude oil concentration. Alternatively, the membrane filtration can be used for preliminary concentration, followed by other processes for handling the preconcentrated feed to achieve higher permeate recoveries.

## Figures and Tables

**Table 1:** Properties of membrane samples.

Membrane	Pore Diameter (µm)	Clean Water Permeability (L/(m^2^ h bar))	Thickness (µm)	Contact Angle (°)
PSF/PEG-0	0.126	329 ± 7	218 ± 1	70.3 ± 0.6
PSF/PEG-30	0.057	365 ± 7	234 ± 1	67.1 ± 0.5
PSF/PEG-60	0.032	502 ± 9	235.7 ± 2	57.7 ± 0.6

## Data Availability

The data presented in this study are available on request from the corresponding author.
